# Contextual factors affecting integration of eye health into school health programme in Zanzibar: a qualitative health system research

**DOI:** 10.1186/s12913-023-10469-9

**Published:** 2023-12-14

**Authors:** Ving Fai Chan, Elodie Yard, Eden Mashayo, Damaris Mulewa, Lesley Drake, Fatma Omar

**Affiliations:** 1 Centre of Public Health, School of Medicine, Dentistry and Biomedical Medicine, Royal Victoria Hospital, Institute of Clinical Sciences, Queen’s University of Belfast, Block B, Belfast, BT12 6BA UK; 2 Brien Holden Vision Institute Foundation Africa Trust, Durban, South Africa; 3 University of KwaZulu Natal, Durban, South Africa; 4 Partnership for Child Development, Imperial College London, London, UK; 5 Oriole Global Health, Nairobi, Kenya; 6 Ministry of Health, Zanzibar, Tanzania

**Keywords:** School eye health, Integrated, Nutrition, FRESH, Vision

## Abstract

**Background:**

Short-term school eye health programmes supported by external funders have sustainability issues. This study aimed to understand the contextual factors affecting integrating eye health into the school health programme.

**Methods:**

We elicited responses from 83 respondents, purposefully selected from the Ministry of Health (*n* = 7), Ministry of Education and Vocational Training (*n* = 7), hospitals/eye centres (*n* = 5), master trainers (4) and schools (*n* = 60) who participated in in-depth interviews. Their responses were analysed and grouped into contextual factors according to the WHO Consolidated Framework for Implementation Research: stakeholders/political, institutional, physical, cultural, delivery system and others. Themes were then generated, and quotations were presented to illustrate the findings.

**Results:**

The six contextual factors affecting the integration of eye health into the school eye health programme were i) Stakeholders/political (Good ministry coordination, defined departmental roles and resource mobilisation from multiple stakeholders; Good stakeholder synergies and address current gaps); ii) Institutional (Institutional coordination and adequate clinic space; Securing human and financial resources; Strategic advocacy for institutional resources); iii) Physical (Long travel distance to service points); vi) Cultural (low eye health awareness among parents, teachers and children); iv) Delivery system (Practical approach to increase screening coverage using teachers as screeners; Balance teachers’ workload, increase screening sensitivity and follow up and; v) Others (Comprehensive training material and effective training delivery; Improved curriculum, teacher selection and supervision and incentives).

**Conclusion:**

Integrated school eye health delivery is generally well-received by stakeholders in Zanzibar, with the caveat that investment is required to address the six contextual factors identified in the study.

**Supplementary Information:**

The online version contains supplementary material available at 10.1186/s12913-023-10469-9.

## Background

Poor health is associated with 200 to 500 million lost school days annually in low-income countries [1]. Some of the most common health conditions, including poor vision, negatively affect educational performance among school-age children [2]. Furthermore, poor child eye health is closely associated with low self-esteem, poor cognitive abilities [3, 4], reduced quality of life, and reduced future economic productivity [5, 6]. However, the prevalence of childhood blindness is high –an estimated 8,500 to 10,000 blind children live in East Africa [7] and Zanzibar alone, and 42% of the children in rural communities who need it did not have a pair of glasses [8].

Recognising the importance of good vision, many countries have made eye health an essential part of school health programmes. In low- and middle-income countries (LMICs), these programmes are usually implemented vertically outside of the public health systems with non-governmental organisations (NGO) support. Given the short-term funding for these programmes, these vertical approaches do not strengthen the local health systems and limit long-term programme impacts. Despite the great need for integration, the evidence of integrating eye health into mainstream school health programmes to ensure effectiveness and efficiency is currently weak [9, 10].

Zanzibar's healthcare system is hierarchical, and organised into three levels of care—national, district and health facility. The District Health Management Team coordinates all health services at the district level and refers cases to the health facility and national level [11]. In terms of eye care, child eye health services, such as basic eye examination, eye drops distribution and refraction services, are provided at all levels of health care at primary health care units, regional hospitals and the national referral hospital. To improve service uptake at the service points, ad hoc- school eye health programmes were conducted since the early 2000s as eye health outreach programmes. These programmes in Zanzibar, which usually cover 20 to 30 schools (coverage 10 – 15%) in densely populated areas, train teachers to conduct a simple visual acuity screening to identify children with reduced vision and obvious eye health diseases usually red eyes and cataracts for treatments. Spectacles, antibiotic eyedrops and surgery were provided free to the children. For example, from 2012–2016, eye health outreach programmes were conducted with NGO support. Despite achieving high screening and treatment rates, the programme had to end when the funding ceased (Zanzibar Eye Health Project, 2017, unpublished).

Recognising the need for practical intervention by NGOs and ensuring the school eye health programme’s sustainability, the Zanzibar Government seeks to integrate eye health into the school health programme. While the current school health programme focuses on water, sanitation, food, and nutrition, the Revolutionary Government of Zanzibar identified that public health practices and access to disability-related services should be improved (Eye Health Strategic Plan 2018 – 2022, unpublished). Hence, the Government aims to integrate school eye health with the school health programme, aligning with the aim of Focusing Resources on Effective School Health, A FRESH Approach for Achieving Education for All *“to implement school-based health programmes in efficient, realistic and results-oriented ways”* [12]. The FRESH approach is adopted by the government and the NGOs working in school health in Zanzibar as it inter-agency framework that guides school health programmes in low-resource settings.

An implementation research to compare the performance of an integrated and vertical school health eye programme was collaboratively conducted by the Ministry of Health and local key stakeholders, an eyecare non-governmental organisation and a child health and nutrition research and technical assistance group from April to October 2017. In the integrated model of delivery, eye health was integrated into mainstream school health programmes where a nutrition programme already exists whereas in the vertical model of delivery, eye health is offered as a stand-alone intervention by an NGO. It was found that the integrated model achieved 96% screening coverage, the cost per child screened was only $1.23, and the cost per child identified as having an eye problem in the integrated model was only half that of the vertical model ($24.76 vs $51.75) [13]. Further, when compared with the vertical model, the integrated model achieved better reach, effectiveness, adoption rate, and implementation performance [14]. But, both models were discontinued when the funding ended [14]. Hence, the authors recommended an integrated model to ensure the sustainability of school eye health delivery.

Subsequently, a series of in-depth interviews using a systematic approach was conducted. The objective of the research was to discuss the implementation stakeholders’ (Ministry of Health, Ministry of Education, hospitals/eye centres, master trainers and school representatives) to understand the contextual factors that can influence the integration of school eye health into the school health programme.

## Methods

This study’s data was collected as part of the larger implementation research to build the evidence base for an effective school eye health intervention in Zanzibar (the parent study). The study consisted of a quantitative study to compare the performance and costs of an integrated and vertical school health eye programme [13]; and a qualitative study to obtain the partners’ views on the future implementation of the integrated school eye health programme and how to realise this. This article focuses on the qualitative study. We prepared this paper according to the Consolidated Criteria for Reporting Qualitative Research (COREQ): a 32-item checklist for interviews and focus groups.

Two qualitative interviewers with social science backgrounds (male and female) were trained to conduct the in-depth interviews. Both researchers have more than ten years of experience implementing school health projects in Tanzania, including integrating health initiatives into the mainstream health system.

### Interview respondents- sample and composition

The 83 respondents, purposefully selected from the Ministry of Health (*n* = 7), Ministry of Education and Vocational Training (*n* = 7), hospitals/eye centres (*n* = 5), master trainers (4) and 19 schools (*n* = 60), participated in the study. These respondents were selected because they were involved in the delivery of the integrated school eye health programme in the parent study, hence have good understanding of the concept of integration, relevant experience and knowledge in eye health and thus could provide rich and diverse information and practical recommendations on integrating eye health into the school health programme in Zanzibar. The interviews were conducted at the respondents’ offices with no third party present to ensure that they were comfortable giving their responses. Table 1 shows the composition of the respondents.

**Table 1:** The composition and numbers of the respondents who participated in the in-depth interviews

Respondents (number)	Composition
Ministry of Health interviewees (*n* = 7)	Director-General, Director of Planning, Policy and Research. Director of Preventive Service and Health Promotion, District Medical Officers from Micheweni, Mkoani, North A and Unguja districts
Ministry of Education interviewees (*n* = 7)	Director, Department of Policy and Planning, Deputy Principal Secretary, Director for Pre-primary and Primary Education, District Education Officers from Micheweni, Mkoani North A and Unguja
Hospitals/eye centres interviewees (*n* = 5)	Three optometrists from Mjini Magharibi, one optometrist from Chake Chake District Hospital and one optometrist from Mnazi Mmoja Hospital
Master trainers (*n* = 4)	Two ophthalmic clinical officers and two Home Grown School Feeding Programme officers
School interviewees (*n* = 60)	Twenty headteachers and forty school health teachers

### Data collection

The interviews were conducted using an in-depth interview guide designed in discussions with different local implementing stakeholders and tested in a smaller group to ensure the guide’s content and wording were appropriate. We designed the study by asking the overarching question, “How can we integrate eye health into the existing school health programme?” with probe questions that aimed to explore their opinion in a more in-depth manner. The interviews took 45 to 60 min and were audio-recorded with field notes made during the interviews. The interviews were not repeated because it was challenging to schedule interviews with our respondents who had busy working schedules. Instead, debriefing sessions were conducted with the respondents after the interview to make corrections or add additional comments to the notes.

### Data analysis and reporting

Each interview was transcribed verbatim, comprehensively reviewed, and coded by two data coders (RK and MM). An MS Excel database was created to capture the meaning units and display the systematic relationships between coded texts. The data coders linked the meaning units from the transcripts to similar statements across interviews. To explore the data and conceptualise the findings, related ideas across the interviews were located by bringing together strands of data. Subsequently, the data coders generated the themes and attempted to consolidate them while referring to the analytic framework of WHO Contextual Factors in Implementation Research [15], which includes stakeholders, health system, socio-economic, cultural, political, physical, institutional and others. Quotations are presented to illustrate the findings. As the number of respondents in some categories was small, it was impossible to anonymise their identities. Hence, we assigned the respondents from the different categories into i) the Ministry of Health as MOH 1 to 7; ii) the Ministry of Education and Vocational Training as MOEVT 1 to 7; iii) hospital optometrists as Optom 1 to 5; iv) master trainers as MT 1 to 4; v) headteachers as HT 1 to 20; vi) teachers as TCH 1 to 40. The themes and example quotes from the qualitative interviews are shown in Table 2.

**Table 2:** Themes and example quotes from the qualitative interviews

Contextual factor	Quote (Respondent number)
Stakeholders/Political	1. Generally, we should strengthen health promotion as a unit, then Human resources, infrastructure, Budget, and IEC materials in eye care can come but my emphasis is integrating all aspects of health promotion. (MOH 3)
	2. We need to increase awareness among the teachers and parents about the children’s eye health (TCH 41) 3. Children’s eye health awareness (has to be improved) and they can be an ambassador to parents at home (TCH 14)
	4. District authorities to be involved in planning to get a clear picture of what needs to be done. (MOH 7)
	5. Everyone involved needs to meet and plan together. (HT 1) 6. There is a need to frequently visit the schools to identify the challenges and how can be solved (TCH 11)
Institutional	7. There is great success in integration. We have done that, and we have seen the results. Good coordination is strength. We have supported each other in terms of staff and other resources in the integration of Reproductive health and other health interventions. (MOH 1)
	8. It [the integrated programme] has worked well and will continue to work well if we make sure all who are involved make sure that students are screened to know the children with eye problems are referred to the hospital for more treatment. (TCH 9)
	9. One critical aspect is to create adequate clinical space to serve the child patients. This will need coordination from both Ministries. (MOH 3)
	10. There is no budget for integrated School Eye Health Programmes at the district level, but there is at the Regional Government level. A decision to decentralise the three sectors of agriculture, health (basic health at the health centres) and education (nursery and primary), has been made. (MOEVT 7)
	11. The best way we can combine efforts with eye health is by having a strategic plan specific to eye health and having a road map that will lead to different interventions in eye care, then identifying human and financial resources and how they will be utilised. (MOH 2)
	12. (We need to) prepare enough budget and motivate the ministry and stakeholders for funding. (TCH 12)
	13. Yes, but this has to be strongly accepted by the policymakers, and there should be proof of the need/problem existing which indicates that it is worth investing in. (MOH 4)
	14. The clinic is so far from here [schools], only in the town centre. (TCH 49)
	15. It’s so far and transport is not frequent and more expensive. (HT4)
Physical	16. According to me, primary eye care units must first be strengthened, and equipped with enough staff because that is the first referral point from schools. But all this will be achieved if we have a specific road map for eye care. (MOH 1)
Cultural	17. Lack of awareness of both school children and parents, low supervision at school level and lack of collaboration between parents and teachers. (TCH 39)
	18. Stigmatised by friends/schoolmates, the unwillingness of the pupil, parental support lacking. (HT 14)
Delivery system	19. This is a good initiative. I feel like I have contributed to the welfare of the children. Education goes in line with the ability to see well and not have vision problems. If you cannot see well, it affects your learning process. It is a good way to get early identification of childhood blindness and vision impairment. (MT 1)
	20. It saves a lot of time. We spend almost 8 h a day with them. It’s practical even though it is not easy, because of time constraints and a high number of pupils. (HT6)
	21. This is a good initiative, and helps to get early identification of vision impairment in children; however, there is a need to simplify some of the work. (MT3)
	22. This is like a supermarket approach. Workplan and services can be obtained from different sectors/partners/interventions at the same time. It saves time and cost—MOH 5
	23. We are afraid that there is time interference between screening and lessons with the few trained teachers. (TCH 11)
	24. Some parents just do not bring their children to the vision centre. (MT 2)
	25. Some referred pupils did not have any visual problems. They did not necessarily need any referral. Some children were emmetropic, but they (the teachers) failed to take accurate visual acuity. (Optom 1)
Others	26. The training materials were sufficient. (TCH 39)
	27. Very participatory. Teachers understood through a combination of approaches employed such as lectures, group discussions, plenary presentations and practical exercises on vision screening of peers. (MOEVT 2)
	28. The time was short, and a lot had to be covered within a short time. It was quite a challenge for teachers to catch up with the speed and content of the training. (MT 4)
	29. Consider teacher’s age when selecting teachers to be trained, increase training time, conduct supportive supervision, provide incentives for teachers during implementation, and train teachers to train other teachers when they return to school. (MT 2)

## Results

Six contextual factors affecting school eye health integration were identified including stakeholders/political, institutional, health system, physical, cultural and others. Altogether eleven themes were obtained.

### Stakeholders/political

#### Theme 1: Good ministry coordination, defined departmental roles and resource mobilisation from multiple stakeholders

The integration requires multi-stakeholders’s involvement and coordination, with defined roles and responsibilities (Table 3). The five stakeholders that would be responsible for the implementation are the Ministry of Health (MOH), Ministry of Education and Vocational Training (MOEVT), Preventative Service and Health Promotion Unit (PSHPU), District Health Office (DHO) and the District Education Office (DEO). While MOH is responsible for the overall programme and implementation at clinical service sites, MOEVT will facilitate activities at the school level. MOH and MOEVT will be supported by DHO and DEO respectively. The main role of PSHPU would be the development and integration of eye health education into the programme. All stakeholders are to be involved in the monitoring and evaluation of the programme.

**Table 3:** Perceived departmental roles and responsible activities in school eye health implementation

Department	Roles	Responsible activities
Ministry of Health	• In charge of primary health care, tertiary health care (primary eye care based in the community) • Outreaches/camps in schools; monitoring, health promotion and education	• School health monitoring programmes in schools
Ministry of Education and Vocational Training	• Design and coordinate support for school children with health problems • Supervision of schools involved in the school health programme	• Supervision of teachers to implement the school health programme effectively • Training of teachers on school health
Preventative Service and Health Promotion Unit	• Coordinate health promotion and education activities, and service provision	• School health boards to initiate and support health promotion activities within schools
District Health	• Implement health promotion activities • Primary health care, monitoring, school hygiene and immunisation programme	• Create awareness of and monitor health promotion activities
District Education	• Supervise and monitor the school health programme at the district level	• Training of schoolteachers and community members • Setting up management information systems • Resource mobilisation

#### Theme 2: Good stakeholder synergies and address current gaps to ensure eye health is successfully integrated into the school health programme

The synergies among the stakeholders that could improve the implementation of the integrated school eye health programme include i) identifying areas highly affected by eye health problems, ii) planning, implementing and monitoring activities, and addressing operational challenges, iii) coordinating the implementation of teachers training and iv) implementing health promotion activities, which includes community awareness programmes. (Quotes 1 to 3) On the other hand, gaps that need to be addressed were that i) school eye health is not mainstreamed; hence it is not budgeted for and ii) district authorities are not involved in planning, and thus there is a lack of proper coordination between regional and district level (Quote 4). Teachers highlighted that there is a need to involve them in the planning and frequent visits to the schools to solve challenges in the integration process (Quotes 5 and 6).

### Institutional

#### Theme 1: Institutional coordination and adequate clinic space

Respondents from the MOH and MOEVT saw the advantage of integration as the efficient use of resources. They highlighted that unity and coordination are key to successful integration, (Quote 7) and was agreed by teachers (Quote 8). The optometrists and the MOH respondents believed that integrating eye health into the school health programme could be achieved if coordination and clinic space challenges were overcome. (Quote 9).

#### Theme 2: Securing human and financial resources

A major challenge the MOH and MOEVT foresaw is the inadequate budget allocation for the school eye health programme. (Quote 10) The MOH recognises that integrating eye health into the school health programme requires a clear roadmap and resources for implementation. However, identifying and allocating human and financial resources was also emphasised as the main challenge in the process. (Quote 11) This was also observed by the schools where they recommended that a budget should be prepared to apply for funding from the ministry and other stakeholders (Quote 12).

#### Theme 3: Strategic advocacy for institutional resources

The primary resources for maintaining the integrated school eye health programme were the government budget, development partners (community-based organisations, faith-based organisations, non-governmental organisations), community health workers and health centres. It was also pointed out that funding, albeit limited due to competing health priorities, exists in the health care budget to implement and maintain the integrated school eye health programme. Access to the funding depends on the ability to show that an integrated school eye health programme is necessary and cost-effective. (Quote 13).

### Physical

#### Theme 1: Long travel distance to service points

The respondents felt that district primary care units could be service points for children to access eye health as vision centres do not exist in all districts. Headteachers and teachers reported that parents and children need to travel long distances using under-developed public transport systems to access available eyecare services in bigger cities (Quotes 14 and 15). However, these primary care units must be well-equipped, and the staff must be upskilled to handle eye-related issues. (Quote 16).

### Cultural

#### Theme 1: Low eye health awareness among parents, teachers and children

The main concern from the respondents was regarding the children’s low spectacle wear. They stated that parents and teachers do not encourage their children to wear their spectacles. (Quote 17) Teachers have observed the peer-teasing of children who wear spectacles and associated this with the low awareness of the importance of eye health among children and parents. (Quote 18).

### Delivery system

#### Theme 1: Practical approach to increase screening coverage using teachers as screeners

Most of the respondents indicated that eye health screening conducted by teachers was a good initiative and worked well. The teachers felt their contribution to identifying and managing children with eye health problems was recognised. (Quote19) The teachers also felt that conducting eye health screening for children is a practical approach as they spend much time with the students in the schools (Quote 20). The MOH, MOEVT and optometrists further commented that the approach simplifies their work and increases screening coverage. (Quotes 21 and 22).

#### Theme 2: Balance teachers’ workload, improve screening sensitivity and follow up

The teachers mentioned strongly that screening high numbers of students interfered with teaching schedules (Quote 23). Furthermore, some children were afraid to be screened, and children who failed the eye health screening did not go to the hospital for further management. (Quote 24) Optometrists further highlighted the issue of teachers referring children without eye problems to the hospital. (Quote 25).

### Others

#### Theme 1: Comprehensive training material and effective training delivery

The trainers and teachers were satisfied with the training. Almost all the teachers felt that the training materials had enough information (Quote 26). The trainers also commented that the training materials were very clear and could be easily understood. Both trainers and teachers responded that the training, which consisted of group discussions, class participation and lectures, was delivered in a participatory and inclusive manner. (Quote 27).

#### Theme 2: Improved curriculum, teacher selection and supervision and incentive

Most of the respondents felt that the length of time allocated for the training (two days for the integrated model) was insufficient. The trainers felt there was not enough time to cover the training’s content. (Quote 28) Hence, they suggested increasing training days and focusing on teacher selection, supervision, and incentives to improve training outcomes. (Quote 29) The trainers and the MOH respondents felt that training for different topics should be conducted separately to avoid confusion, overburdening the teachers and time constraints.

## Discussion

**Fig. 1:**
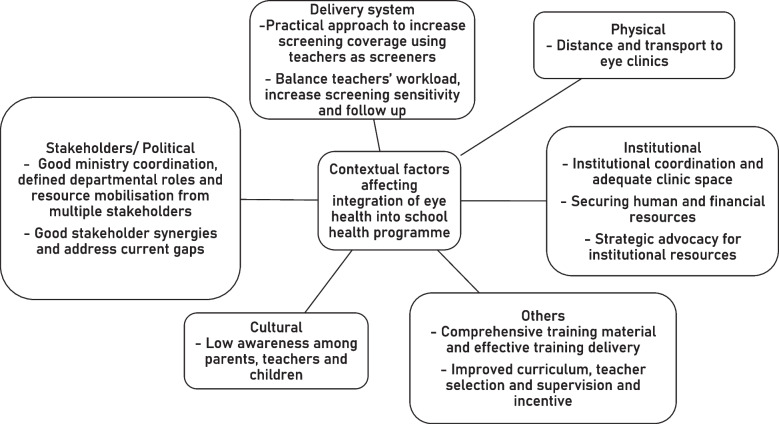
Contextual factors affecting the integration of eye health into the school health programme

While stakeholder engagement is a cornerstone for any health programme integration, there is very little published evidence on this topic. We attempted to methodologically understand the contextual factors that could affect the integration of eye health into the school health programme in Zanzibar. These contextual factors covered stakeholders/political, institutional, physical, cultural, delivery system and other factors. Figure 1 summarises these factors.

Both stakeholders/political and institutional level factors to eye health integration revolved around three main issues – good coordination among stakeholders, gaps identification, and human and financial resources mobilisation. Based on the detailed roles and responsibilities, synergies and gaps identified, there is clear commitment, good leadership and governance: all positive catalysts for integrating eye health into the school health programme. Furthermore, currently, eye health services are delivered through the MOH, while school health programme is coordinated through the MOEVT. Our findings are imperative as there seems to be agreement on the need to share responsibilities in delivering integrated school eye health in Zanzibar among the local implementers. Even though published examples of leadership and governance in policy setting and implementation, leading to quality care, are limited in primary eye care [16], we have observed in this study was there is a clear identified pathway towards integration. The first step in integrating eye care is health system planning to create and enable an optimal environment for integration [17].

Participants also highlighted the need for synergies and identifying gaps in evidence in the burden of eye health, operational challenges, coordination of teachers’ training and issue of the lack of health promotion strategies locally. It must be mentioned that our work was the first larger-scale implementation research that provided findings on the prevalence of eye disease among children in Zanzibar [13, 14] and identified operational challenges that influenced the project delivery such as interruption by rainy seasons and delay of starting of screening due to screening schedule interrupt with teaching [14]. All these findings are utilised to further revise an effective and efficient integrated school eye health programme in Zanzibar.

The lack of human and financial resources dedicated to eye care is identified as a barrier to integration. Since eye health is currently not a national school health programme component, there is no specific budget dedicated to the school eye health programme. This is a common challenge faced by LMICs [18]. While the Government can allocate resources for school eye health, continuous advocacy must ensure its integration with the national school health programme. Following the study’s completion, multiple stakeholder discussions have been held with local government ministries, non-governmental organisations, local stakeholders and beneficiaries, forming a child eye health forum to find solutions to mobilise resources for school eye health in Zanzibar.

Another institutional-level factor is to ensure the availability of clinic space to address the school screening referrals. A sudden increase in patients attending the vision centres following teachers’ referrals was a major challenge. Planning projections before the pilot proved too conservative as the vision centres could not cope with the sudden surge in patient loads. Our pilot provided valuable information for realistic planning to ensure high-quality care [19, 20].

The primary physical factor pointed out repeatedly was the long distance from schools to the clinics, especially in town areas. The second and third strategies of Integrated People-centred Eye Care (IPCEC) [17] emphasises that eye care should be reoriented towards prioritising services delivered at the primary and community level. The aim is for those families who live further away from the vision centres not to be deprived of access to services. It is encouraging that the respondents suggested providing eye care services at the primary health units closer to the communities than the vision centres and private sector optical outlets. Currently, discussions are also ongoing with the MOH to include eye health services at primary health units and that eyecare treatment for children would be covered under the National Insurance Scheme.

One main cultural factor that would influence service and treatment compliance is the awareness of teachers, parents and children towards eye health. The respondents’ repeated suggestion was to improve the existing eye health education strategy to increase spectacle usage and compliance. There was minimal investment in child eye health promotion in Zanzibar. However, this is a worldwide phenomenon where attention is focused on treatment. However, health promotion activities have shown to be effective in improving eye health knowledge and awareness in the community, among the older population and those with diabetes, and increasing the uptake of eye services in Bangladesh [21]. Furthermore, the development of an innovative arts-based child eye health education strategy has been completed [22] and will be piloted in 2024. The arts-based eye health education strategy aims to use traditional and contemporary music to convey eye health messages to teachers, parents and children to improve eye health services uptake.

In terms of delivering the integrated school eye health programme, the respondents agreed that training teachers in eye health screening is essential, given the high teacher-student contact time. This teacher-led screening approach aligns with Tanzania’s National Eye Health Strategic Plan 2018–2022 and the IPCEC Strategy [17] to empower and engage communities in providing eye care to children. Empowering communities has improved early disease detection, timely intervention, and compliance. Furthermore, successful task-sharing by extending responsibilities to lay personnel has been shown to increase programme effectiveness [23]. Similar findings were obtained from the scaling-up assessment of the school eye health programme in Zambia [24], where task-sharing will play a critical role in ensuring the successful integration of eye health into the school health programme.

In general, the respondents were satisfied with the training and the training material. To ensure the programme’s effectiveness and efficiency, they suggested more teachers should be trained, with refresher training conducted at regular intervals to maintain and improve the screening quality. Where human resource training was ineffective, there is evidence to show that it was due to inadequate content and quality of training and a lack of support to implement the eye health skills learned [25–29]. We also recommend that further supervision, ongoing motivation and support are provided.

While the teachers in our programme were willing to learn the additional eye health screening skills, caution must be taken to ensure teachers understand that eye health is part of child health so that performing eye health screening is not perceived as an ‘extra’ duty. Hence, it is also critical that the stakeholders understand that the aim is not to train teachers to become eye specialists but to accurately detect children with eye health problems and refer them sufficiently early [25, 30].

The limitations of the study must be acknowledged. The study was conducted in Zanzibar where the population is small, with a well-established healthcare system despite under-resourced. Hence, our study findings must be interpreted carefully within this context. The study only included implementers as participants in the interviews and parents and children’s opinions have been excluded.

## Conclusions

The study identified six important contextual factors that affect the successful integration of eye health into school health programmes for inclusion in the National Health Policy, with the caveat that investment is required for ensuring good coordination, advocacy for resource mobilisation, addressing operational challenges and physical and cultural barriers and community sensitisation.

### Supplementary Information

**Additional file 1.** Qualitative interview transcripts. The qualitative interview transcripts are obtained from interviewing 83 stakeholders in Zanzibar.

## Data Availability

Included in the Supplementary material.

## Abbreviations

NGO: Non-governmental organization
LMICs: Low and middle-income countries
FRESH: Focusing Resources for Effective School Health
COREQ: Consolidated Criteria for Reporting Qualitative Research
MOH: Ministry of Health
MOEVT: Ministry of Education and Vocational Training
MT: Master trainer
Optom: Optometrist
HT: Headteacher
TCH: Teacher
PSHPU: Preventative Service and Health Promotion Unit
DHO: District Health Office
DEO: District Education Office
IPCEC: Integrated People-Centred Eye Care

## References

[1] Drake L, Jukes MCH, Bundy D (2008). School health, nutrition and education for all.

[2] Grantham-McGregor S, Cheung YB, Cueto S, Glewwe P, Richter L, Strupp B (2007). Developmental potential in the first 5 years for children in developing countries. Lancet.

[3] Atkinson J, Anker S, Nardini M (2002). Infant vision screening predicts failure on motor and cognitive tests up to school age. Strabismus.

[4] Roch-Levecq A-C, Brody BL, Thomas RG, Brown SI (2008). Ametropia, preschoolers’ cognitive abilities and effects of spectacle correction at 6-month follow-up. Arch Ophthalmol.

[5] Joy S, Frick K, Naidoo K, Wilson D, Holden B. The global burden of potential productivity loss from presbyopia. In: Investigative Ophthalmology & Visual Science. Maryland: C.V. Mosby Co; 2013. http://iovs.arvojournals.org/article.aspx?articleid=2149415. Accessed 10 Jan 2018.

[6] School Health Programme Advocacy Paper - IAPB. https://www.iapb.org/resources/school-health-programme-advocacy-paper/. Accessed 16 Jul 2020.

[7] Gilbert C, Awan H (2003). Blindness in children. Br Med J.

[8] School Health Integrated Programming. Sightsavers. https://www.sightsavers.org/programmes/school-health-integrated-programming/. Accessed 29 Apr 2020.

[9] Keugoung B, Macq J, Buvé A, Meli J, Criel B (2011). The interface between health systems and vertical programmes in Francophone Africa: the managers’ perceptions. Trop Med Int Heal.

[10] Reich MR, Takemi K (2009). G8 and strengthening of health systems: follow-up to the Toyako summit. Lancet.

[11] Ministry of Health Zanzibar. Zanzibar guidelines for Integrated Disease Surveillance and Response (IDSR). https://mohz.go.tz/eidsr/. Accessed 17 Feb 2018.

[12] Focusing Resources on Effective School Health: A FRESH Approach for Achieving Education for All. Improving learning outcomes by improving health and nutrition: Incorporating the FRESH approach in National Action Plans for achieving EFA. http://www.unesco.org/education/tlsf/mods/theme_b/img/FRESH.pdf. Accessed 25 Oct 2017.

[13] Chan VF, Omar F, Yard E (2021). Is an integrated model of school eye health delivery more cost-effective than a vertical model? An implementation research in Zanzibar. BMJ Open Ophthalmol.

[14] Chan VF, Yard E, Mashayo E, et al. Does an integrated school eye health delivery model perform better than a vertical model in a real-world setting?: A non-randomised interventional comparative implementation study in Zanzibar. Br J Ophthalmol. 2022. 10.1136/bjo-2022-321752. 10.1136/bjo-2022-321752 PMC10804016 36376063

[15] Implementation research toolkit. https://apps.who.int/iris/handle/10665/110523. Accessed 27 March 2023.

[16] Dieleman M, Shaw DMP, Zwanikken P (2011). Improving the implementation of health workforce policies through governance: a review of case studies. Hum Resour Health.

[17] World Health Organization. World report on vision. 2019. https://www.who.int/publications/i/item/world-report-on-vision.

[18] Backman G, Hunt P, Khosla R (2008). Health systems and the right to health: an assessment of 194 countries. Lancet.

[19] Reerink I, Sauerborn R (1996). Quality of primary health care in developing countries: recent experiences and future directions. Int J Qual Heal Care.

[20] Lindfield R, Vishwanath K, Ngounou F, Khanna RC (2012). The challenges in improving outcome of cataract surgery in low and middle income countries. Indian J Ophthalmol.

[21] Islam FMA, Chakrabarti R, Dirani M (2014). Knowledge, attitudes and practice of diabetes in Rural Bangladesh: the Bangladesh population Based Diabetes and Eye Study (BPDES). Sen U, ed. PLoS One.

[22] Chan VF, Belluigi D, Chee Yong A (2022). Co-creating an arts-based eye health education strategy in Zanzibar: process, outcomes and lessons learnt. BMJ Glob Heal.

[23] Lewin S, Munabi-Babigumira S, Glenton C (2010). Lay health workers in primary and community health care for maternal and child health and the management of infectious diseases. Cochrane Database Syst Rev.

[24] Yong AC, Buglass A, Mwelwa G, Abdallah I, Chan VF (2022). Can we scale up a comprehensive school-based eye health programme in Zambia?. BMC Health Serv Res.

[25] Courtright P, Seneadza A, Mathenge W, Eliah E, Lewallen S (2010). Primary eye care in sub-Saharan African: do we have the evidence needed to scale up training and service delivery?. Ann Trop Med Parasitol.

[26] Byamukama E, Courtright P (2010). Knowledge, skills, and productivity in primary eye care among health workers in Tanzania: Need for reassessment of expectations?. Int Health.

[27] Onakpoya O, Adeoye A, Adegbehingbe B, Akinsola F (2009). Assessment of human and material resources available for primary eye-care delivery in rural communities of southwestern Nigeria. West Indian Med J.

[28] de Wet M, Ackermann L (2000). Improving eye care in the primary health care setting. Curationis.

[29] du Toit R, Hughes F, Mason I, Tousignant B (2011). Facilitating the quality of care in a specialist Pacific ophthalmic nursing workforce. Int Nurs Rev.

[30] World Health Organization (2009). Prevention of avoidable blindness and visual impairment.

